# Optical detection of the magnetophoretic transport of superparamagnetic beads on a micromagnetic array

**DOI:** 10.1038/s41598-020-69757-7

**Published:** 2020-07-30

**Authors:** Dhruv Gandhi, Peng Li, Stefano Rampini, Charlotte Parent, Gil U. Lee

**Affiliations:** School of Chemistry, University College Dublin, Belfield, Dublin 4, Ireland

**Keywords:** Biotechnology, Engineering, Nanoscience and technology, Optics and photonics

## Abstract

Micromagnetic arrays (MMAs) have proven to be powerful tools for controlling the transport and separation of bioanalytes, i.e., they allow bioanalyte-superparamagnetic (SPM) bead complexes of specific size and magnetization to be moved in a synchronized manner that is precisely controlled with the orientation of an external magnetic field. This article presents a laser-photodetector system for the simple detection of individual SPM beads moving on a specific region of an MMA. This system detects the SPM beads through the change in intensity of reflective light as they move from the highly reflective micromagnetics to the supporting substrate. We demonstrate that this opti-MMA system allowed the size, number, and magnetic and optical properties of the SPM beads to be rapidly determined for regions > 49 µm^2^ in size. The response of the opti-MMA system was characterized in several optical configurations to develop a theoretical description of its sensitivity and dynamic range. The speed, low-cost, and sensitivity of this system promises to allow MMAs to be readily applied in in vitro diagnostics and biosensing.

## Introduction

Superparamagnetic (SPM) microscopic beads are a powerful tool for in vitro diagnostics and lab-scale biotechnology separation including the isolation of nucleic acids, proteins, virus, and cells^1–3^. The increased reaction rates and surface area of microscopic bead suspensions are particularly useful for lab on a chip (LOC) applications resulting in improve sampling speed and sensitivity^4^. SPM bead manipulation in microscale devices can easily be performed with localized magnetic fields generated by permanent magnets or electromagnets^5,6^. More generally, SPM beads have been used for a range of essential operations in LOC devices, e.g., transport, separation, sorting, mixing and detection. Demonstrations using SPM beads in LOC devices include DNA extraction^7^, bead sorting^8^, cell separation^9,10^, rare cell isolation^11,12^, fluidic control^13–15^, and biomarker detection^16–18^. The simplicity of these magnetically driven operations is, however, counterbalanced by the tendency of the beads to form aggregates inside a microfluidic device and the risk of mechanical disruption of rare and fragile cells^19,20^.

Microfabricated permanent magnets patterned inside LOC devices provide a means to create strong magnetic field gradients, between 10^4^ and 10^5^ T/m, in a localized volume. Micromagnetic arrays (MMAs) composed of these permanent magnets have been used to capture and control the transport of SPM beads with micron precision, i.e., the combination of the MMA and an external rotating magnetic field generates a travelling magnetic field wave (TMFV) that translates across the MMA at the speed of rotation of the applied field^21–25^. The SPM beads are trapped in the local magnetic field maxima and at low rotational frequencies are transported at the same velocity as the TMFV. In nonlinear magnetophoresis (NLM), the beads travel synchronously with the external field (“phase-locked regime”) until a critical frequency, *ω*_*c*_, is reached, which is approximately 20 Hz for 2.8 mm diameter SPM beads with a saturation magnetization of 11.2 ± 1 Am^2^kg^−1^ when high quality micromagnets are used. When the rotational frequency is higher than *ω*_*c*_ the beads are no longer synchronized with the rotating field, and they enter a “phase-slipping” regime in which they exhibit a non-linear response to the TMFV and their velocity starts decreasing. Further increasing the rotational frequencies results in bead immobilization on the MMA at a frequency *ω*_*i*_, which for high quality micromagnets is approximately 39 Hz^22,23^. The critical frequency is the frequency at which the population of SPM beads is observed to de-synchronize with the external field, and has a direct dependence on the size and the magnetization of the beads. Thus, above *ω*_*c*_ it is possible to observe different bead behavior due to non-uniformities in size and magnetization. The population of beads in the “phase-slipping” regime increases as the rotation frequency increases until at *ω*_*i*_ all beads are in this regime^21^. The local magnetic field of the micromagnets also dominates the bead-bead interactions for moderate bead magnetizations preventing the formation of aggregates and chains^23^. This has allowed MMAs to be used for a variety of precise single-particle operations, e.g., the transport of magnetic beads has been controlled with nm-precision, rapid-multiplexed separation has been achieved in mL-scale volumes, pumping-mixing has been demonstrated in microchannels without external fluidic systems, and automated detection of magnetic beads and biological targets has been performed^24^.

On-chip SPM bead detection for biosensing has been demonstrated using optical microscopy^26,27^, Hall effect^25,28–30^, tunneling magnetoresistance^31^, giant magnetoresistance^32–35^, and biochemiresistor sensors^36^. These technologies are very sensitive but limited in scope by cost, complexity, need for calibration, and limited sensitivity. Recently, the detection of magnetic beads and bioanalyte attachments has been successfully implemented with optical methods^16,37–39^. For example, beads and cells patterned on a specifically functionalized surfaces were detected by comparing the transmitted and diffracted light intensity^38^. This approach, however, requires a high degree of control of the chemistry of the surface, which ultimately limits its application for biosensing. We have found that micromagnets are highly reflective, acting as micro-mirrors, forming the basis for the detection of optically active SPM beads^40^. In this article we demonstrate that SPM beads modulate the reflected intensity of light so that it is possible to extract information about the number, size, velocity and the presence of a biological target. We describe an opti-MMA detection system based on the controlled motion of the SPM beads on the micro-mirrors that is capable of reaching single bead sensitivity through the selection of specific lasers and fields of view (FOV). The sensitivity of this detection system was determined for different NLM operating conditions, i.e., in the phase-locked and phase-slipping mode, and forming the basis for a theoretical model for the response of this detection system.

## Methods and materials

### Detection system set-up

**Figure 1:**
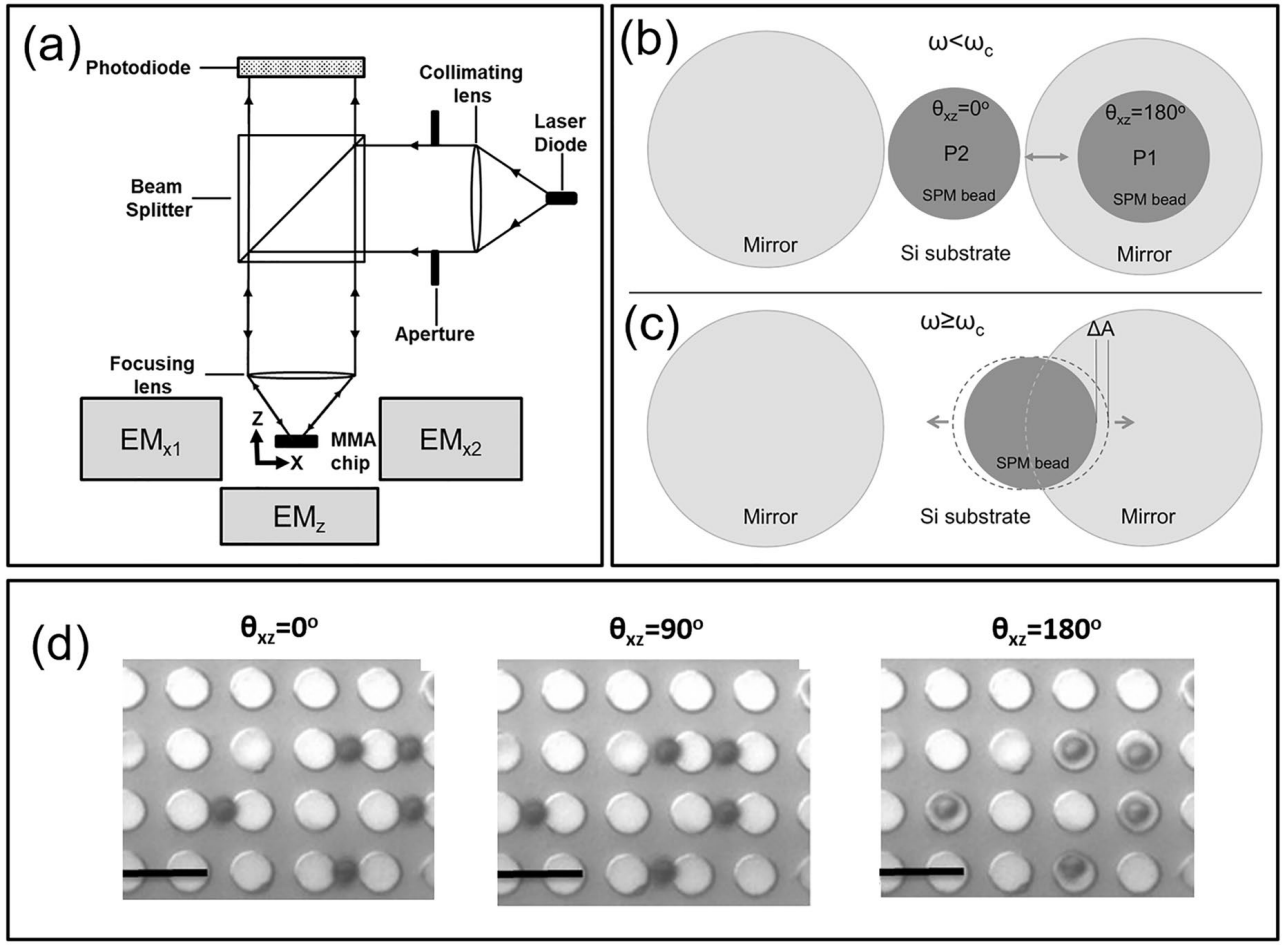
Optical micromagnetic array system and response of SPM beads. (**a**) Schematic of the system that was composed of a collimated monochromatic laser focused on a region of the MMA surrounded by three electromagnets (EM1-3). The electromagnetics produced a rotating magnetic field in the *xz*-plane. (**b**) Schematic of the SPM beads shuttling between two micromagnetics in the phase-locked mode (*ω* < *ω*_*c*_). (**c**) Schematic of a SPM bead oscillating on the edge of a micromagnet in the phase slipping mode (*ω*_*c*_ < *ω* < *ω*_*i*_). (**d**) Optical micrographs of SPM beads on an MMA at different orientations of the external magnetic field (*θ*_*xy*_). Scale bars are 10 µm.

Figure 1a illustrates a schematic of the set-up of the opti-MMA system which is comprised of optical and magnetic components, i.e., the MMA chip, a set of three programmable electromagnets for generating the rotating field in the *xz*-plane, laser source, dichroic mirrors, collimating lens, objective lens, and a photodetector for real-time detection of the reflected light intensity. As described previously, the rotating magnetic field orthogonal to the axis of flow on the F-NLM chip was generated with three electromagnets configured in two axes, as shown in Fig. 1a^22^. The solenoids were composed of 570 coils surrounding a cylindrical iron core (ASTM A536 ductile iron) that had a diameter of 60 mm and length of 150 mm. Two synchronized sinusoidal signals with a 90° phase difference were generated with a two-channel function generator (Tektronix, Beaverton, Oregon, USA). This signal was amplified to the desired current using two programmable amplifiers (Kepco, Flushing, NY, USA) and supplied to the electromagnets assembled in the x and z axes. This generated an elliptical, rotating magnetic field with amplitude of 48 and 29 G in the x direction and z directions, respectively. The uniformity of the magnetic field generated by the electromagnets on the micromagnet array was > 96% in the axial direction and > 83% in the radial direction.

The MMA consists of a periodic array of cobalt micromagnets (2.5 µm in radius, *a*, and 8 µm in center-to-center distance, *d*) on a silicon substrate^22^. A positive photoresist based lift-off process was used to fabricate the cobalt micromagnets with a high degree of dimensional control. The micromagnet array was composed of 5.0 ± 0.1 μm diameter circular magnets arranged in a square lattice with an 8.0 ± 0.1 μm center-to-center distance. The micromagnets were created by thermally depositing 5 nm chromium, 70 nm cobalt, and 5 nm chromium on the patterned photoresist. This thickness of cobalt was chosen to produce the highest values of critical and immobilization frequencies. After lift-off the resulting micromagnets were inspected with scanning electron microscopy and atomic force microscopy to determine their *z* and *xy*-dimensions with a 1 and 10 nm resolution, respectively. The micromagnet arrays were subsequently coated with a 600 nm thick spin-on-glass (SOG) layer (Filmtronics, Butler, PA, USA) to provide chemical protection.

The motion of SPM beads on the MMA was recorded with a high-speed camera (Zeiss Axiocam, Zeiss Gottingen, Germany), as shown in Fig. 1a. Two AlGaInP laser diodes with wavelength of 635 nm (Hitachi HL6322G) and 532 nm (Thorlabs DJ32-40) were used as light sources with a laser diode controller (Thorlabs LDC205C). The divergent laser beam was collimated using a focused variable collimating lens (Linos A240A, Munich, Germany) and then focused on the MMA surface through microscope objectives (Zeiss plan-flour 63×, 0.75 NA or plan-fluor 20×, 0.4 NA). The size of the laser spot on the MMA was adjusted using an aperture inserted in the optical path. By using different wavelengths and objective lenses four different FOVs were created, i.e., a 63× objective produced a 49 µm^2^ (FOV1) and 1,963 µm^2^ (FOV2) illumination area whereas the 20× objective produced a 38,013 µm^2^ (FOV3) and 3.02 mm^2^ (FOV4) illumination area. The light reflected from the MMA was collected onto a high bandwidth silicon photodetector with built-in amplifier (Thorlabs PDA100a, 70 db switchable gain). The output signal from the photodetector was recorded and digitally filtered by using a third order Butterworth filter on the data acquisition card (PCI 6281, National Instruments).

### SPM beads and sample preparation

The commercially available SPM magnetic beads used in this study were 2.8 µm in diameter and coated with a monolayer of protein (Dynabeads M270, Streptavidin, Invitrogen, USA). These beads have been used by this group and others in the study of micromagnetic behavior due to their uniform size, i.e., the coefficient of variation of the radius as < 3%, and defined magnetic properties^41^. The magnetic properties of the M270 beads have been measured using SQUID magnetometry and the saturation magnetization was 11.2 ± 1 Am^2^kg^−1^ and volumetric magnetic susceptibility at fields of less than 1,000 Oe was 0.17^23^. The beads were used as provided at a defined concentration between 10^3^ to 10^6^ bead per mL of phosphate buffered saline with 0.1% of Tween 20 (Sigma Aldrich, Ireland). A thin layer of casein was adsorbed on the MMA surface to minimize attractive forces with the SPM beads. The motion of these beads on the MMA was consistent with previous studies from this laboratory^23^.

### Manipulation and measurement of SPM beads

Figure 1d presents a sequence of microscope images of SPM beads traveling across the MMA as a function of the orientation of the external magnetic field^22^. In this study, we often only used the two x-electromagnets, i.e., EM_x1_ and EM_x2_, producing a magnetic field oscillating in the *x*-direction. In this configuration the beads did not travel across the MMA but rather oscillated from the center of a micromagnet to the center of the region between that micromagnets, as shown in Fig. 1b. When low frequency oscillating magnetic fields were applied, the SPM beads freely shuttle between position 1 (*P1*) and position 2 (*P2*) on the MMA, in the “phase-locked” regime. In the “phase-slipping” regime the SPM beads became immobilized at the edge of the micromagnets, as shown in Fig. 1c. The oscillation amplitude of the beads, *ΔA*, was found to decrease as the frequency of the external magnetic field approached *ω*_c_ until they were immobilized at the edge of the micromagnet when *ω* = *ω*_*i*_^22^. Using this approach, it was possible to use a fixed number of beads simplifying analysis.

The detection process was performed as follows. A 10 µL volume of 10^4^ to 10^7^ SPM beads/ml was placed on the MMA and covered by a glass microscope coverslip. The programmable external magnetic field was activated, and the bead motion was simultaneously monitored with the photodetector and high-speed camera.

## Results and discussion

### Detection of bead transport on the MMA—phase-locked and phase-slipping regimes

**Figure 2:**
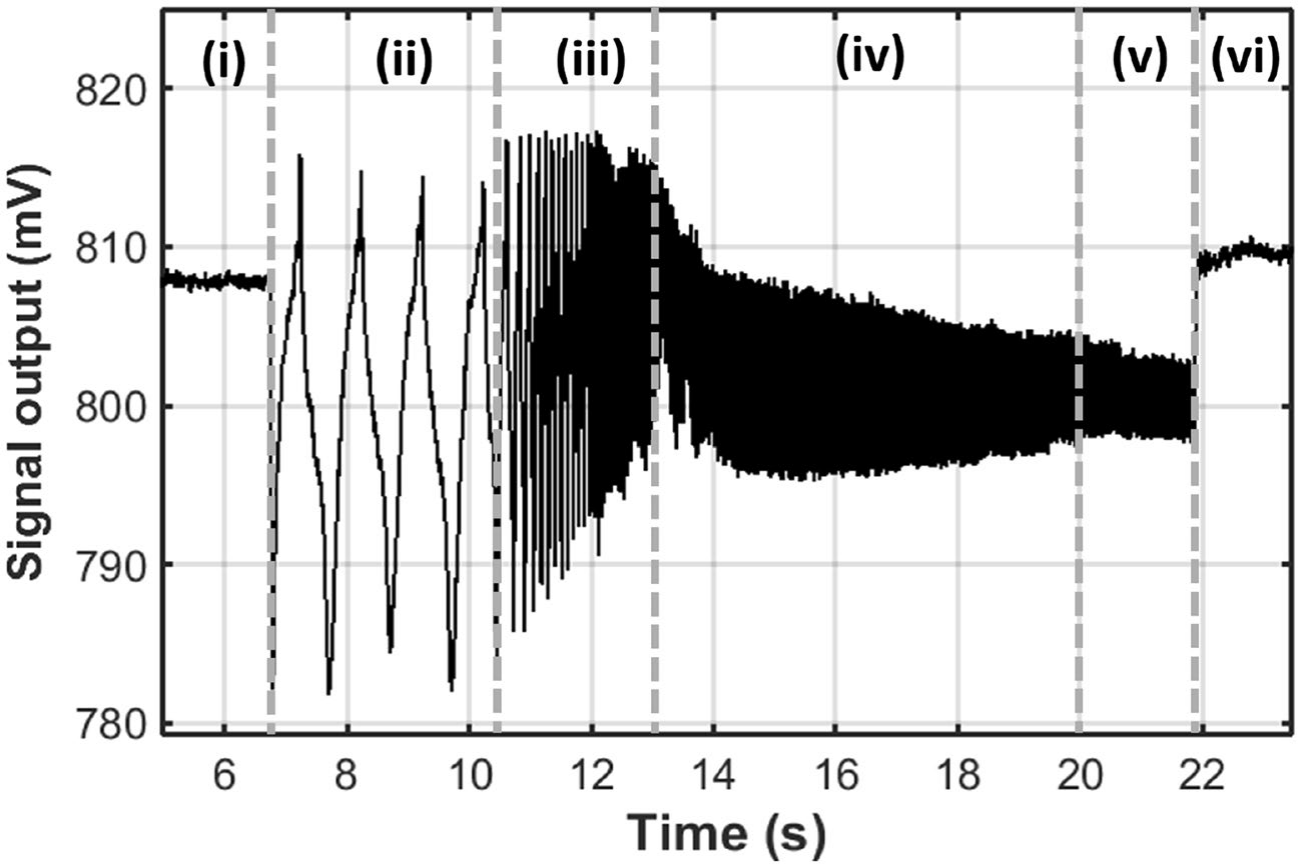
Opti-MMA photodetector signal for a small number of 2.8 µm diameter SPM beads as the frequency of the external magnetic field was increased from 0 to 100 Hz (FOV3). The five labeled regions correspond to external magnetic field frequencies of (i) no external field, (ii) *ω* = 1 Hz, (iii) *ω* < *ω*_*c*_ (2 Hz–19 Hz), (iv) *ω*_*c*_ ≤ *ω* < *ω*_*i,*_(20 Hz–39 Hz), (v) *ω* ≥ *ω*_*i*_(40 Hz–100 Hz), and (vi) no external field. We note that the signal background shifts from *i* to *vi* due to a change in the total number of beads in the FOV.

Figure 2 presents the opti-MMA signal from a region in which SPM beads were dispersed as the frequency of the external rotating magnetic field, *ω*, was varied from 0 to 100 Hz. Five distinct regimes of behavior were identified that are characteristic of the *ω* and the number of the SPM beads. In regions (*i*) and (*vi*) an external magnetic field was not applied to the MMA and the photodetector signal was characterized by a background signal that was set by the number of beads in the optical sensing area and optical properties of the system. In region (*ii*) a low-frequency of rotation of the magnetic field, i.e., *ω* < 19 Hz, was applied to the MMA producing a periodic photodetector signal. In this range of *ω* the beads moved between the micro-mirror and silicon substrate in a phase-locked mode that was synchronized with *ω*, as illustrated in Fig. 1b. Optical imagines revealed that the minima of the photodetector signal (*V*_*min*_ ~ 785 mV) corresponded to all the beads positioned on-top of the micromagnets, *P1* in Fig. 1b, covering the highly reflective chrome regions that acted as micro-mirrors. The peak signal (*V*_*max*_ ~ 815 mV) corresponded to all the beads positioned on the silicon substrate, i.e., between adjacent micro-mirrors, *P2* as shown Fig. 1b. In this position the beads cover the less reflective silicon substrate increasing the total reflected light intensity.

In region (*iii*) the beads motion started to decouple from the external magnetic field at frequency equal to and higher than the critical frequency, *ω*_*c*_. At this frequency, optical microscopy revealed that the beads started to become immobilize on the edge of micro-mirror and oscillated with an amplitude, *ΔA*, as shown in Fig. 1c. The amplitude of the photodetector signal was observed to decrease in region (*iii*), which was a result of the fact that *ΔA* decreased as *ω* increased^22^. As the *ω* increased further it reached the immobilization frequency, *ω*_*i*_, at which all the beads were in a phase-slipping mode and thus immobilized of the MMA. The critical and immobilization frequency of the 2.8 µm SPM beads were determined to be 19 ± 2 Hz and 40 ± 2 Hz, respectively. Region (iv) corresponded to the signal as the frequency was increased from 20 to 39 Hz. In this region, *ω*_*c*_ ≤ *ω* < *ω*_*i*_ and the amplitude of the signal continued decreasing as the oscillation frequency increased. The decreased *ΔA* resulted from an increasing portion of the beads entering a phase-slipping regime. In region (v), *ω* increased from 40 to 100 Hz where *ω* ≥ *ω*_*i*_. In this region, all the beads were in the phase-slipping regime having a minimum oscillation motion at the edge of micro-magnets. This produced an average signal of ~ 800 mV approximately halfway between *V*_*min*_ and *V*_*max*_ with a peak-to-peak frequency ~ 5 mV. This resulted from the fact that SPM beads were trapped in a position between the silicon and micro-mirror, as shown in Fig. 1c.

### Sensitivity of the opti-MMA system: optical configuration and external frequency of rotation of the magnetic field

**Figure 3:**
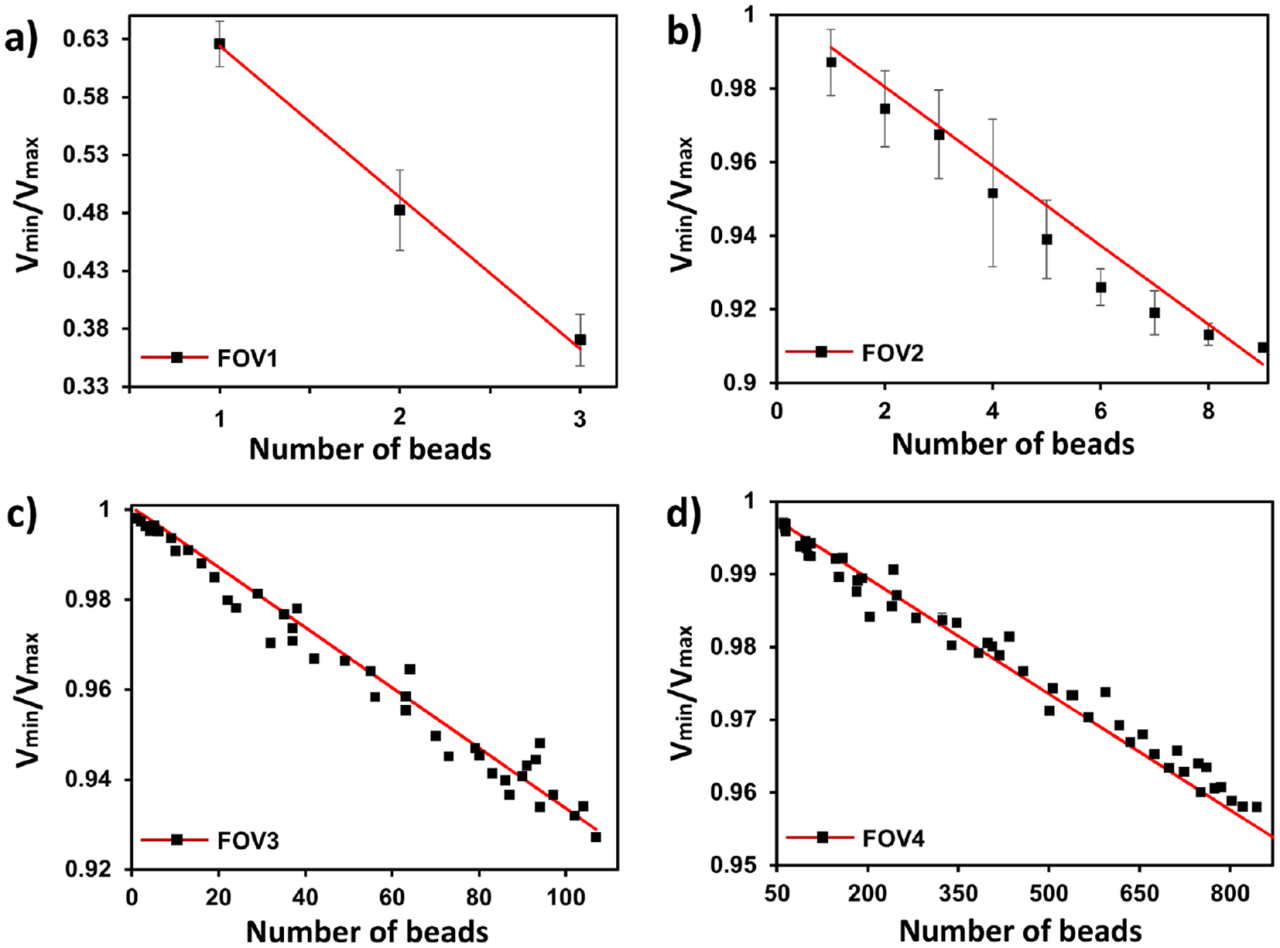
Normalized optical signal versus quantity of SPM beads for different FOV in the phase-locked mode SPM bead transport for (**a**) FOV1, (**b**) FOV2, (**c**) FOV3 and (**d**) FOV4. The redlines present the numerical calculations based on the model presented in “Theoretical analysis of the opti-MMA signal” section. *R*^2^ values for FOV1, FOV2, FOV3 and FOV4 are 0.9949, 0.9753, 0.9752 and 0.9835, respectively. At least three measurements were performed for each data point.

The magnitude of the photodetector signal was studied for four optical configurations, i.e., FOV1, 2, 3 and 4, as a function of the number of the beads in the sensing area. Figure 3 presents the normalized output voltage, *V*_*min*_*/V*_*max*_, for a known quantity of SPM beads for the four optical configurations at a frequency of 1 Hz, which is in the phase-locked transport regime (*ω* < *ω*_*c*_). The relationship between nominalized voltage and the quantity of SPM was similar for all four FOVs, i.e., *V*_*min*_*/V*_*max*_ decreased in a linear fashion with the number of SPM beads in the FOV. The sensitivity, *S*, of the optical detection systems was defined as

1 $$S=\left|{(V}_{min}/{V}_{max})/N\right|,$$

where N is the number of SPM beads in the field of view, was found to be dependent on the optical configuration used to detect the beads. The sensitivity for FOV1, FOV2, FOV3 and FOV4 was 0.1307, 0.011, 0.0007, and 5 × 10^–5^ per bead, respectively. These results suggest that the sensitivity of the detection system was higher for optical systems with a smaller field of view. For instance, a single 2.8 µm bead could be easily detected in FOV1 while the limit of detection was two beads in FOV2, four beads in FOV3, and thirty-two beads in FOV4. Although the sensitivity of detection in larger fields of view was low, they had a wider area of detection and thus were capable of higher throughput, e.g., the numbers of detectable SPM beads in FOV4 varied from 32 to more than 800.

**Figure 4:**
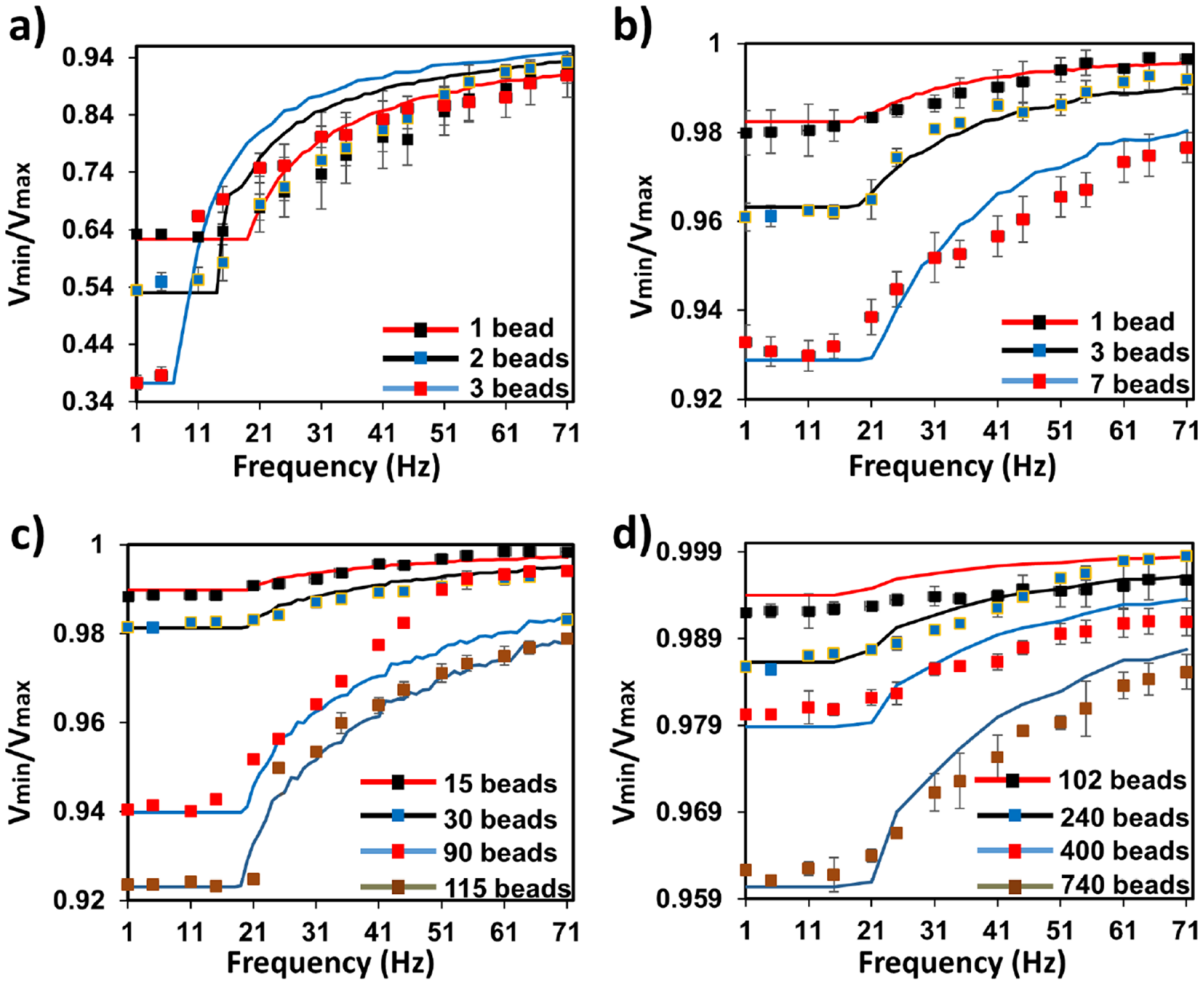
Normalized optical signal versus frequency for different number of beads in four FOVs. (**a**) FOV1, (**b**) FOV2, (**c**) FOV3 for (**d**) FOV4. Each data point presents the mean and standard deviation of three individual measurements. The numerical calculations were based on Eq. (6).

Figure 4 presents the dynamic response of the opti-MMA as a function of *ω* for specific densities of SPM beads in the four different FOVs. For each optical system the *V*_*min*_*/V*_*max*_ was constant at low frequencies for a specific quantity of beads until the critical frequency was reached. This is consistent with the model of SPM beads moving in the phase-locked mode at low frequencies that was described above. The *ω*_*c*_ was found to be 19 ± 2 Hz for all bead and optical configurations except for 3 beads in FOV1. This exception can be attributed to the propensity of the beads to form clusters on a single micromagnet under these conditions. The *V*_*min*_*/V*_*max*_ increased for *ω* higher than critical frequency until it reaches a constant value that was determined by the total number of beads in the FOV. It was also observed that the more beads that were in the FOV the larger the *V*_*min*_*/V*_*max*_ change in the frequency range *ω*_*c*_ < *ω* < *ω*_*i*_. Taken together these results demonstrated that the magnitude of the photodetector signal at a specific frequency could be used to determine the number of beads in the FOV for a specific optical detection configuration.

### Theoretical analysis of the opti-MMA signal

A model has been developed to describe the signal of the opti-MMA detection system for a defined number of SPM beads as a function of the mode of transport. This model is based on describing the change in the photodetector signal as the SPM beads moved from the reflective micromagnets to the supporting silicon substrate, and a *V*_*min*_ and *V*_*max*_ are determined as the beads move in a synchronous manner under the external magnetic in either the phase-locked, *ω* < *ω*_*c*_ , or phase-slipping, *ω* ≥ *ω*_*i*_, regime. In the frequency regime between these two limits, i.e., *ω*_*c*_ ≤ *ω* < *ω*_*i*_*,* the motion of the beads was influenced by variations in their physical properties, i.e., size and magnetization, and the optical signal was a function of *ω*.

The photodetector signal was determined by the optical properties of the system. In the case where there are no beads on the surface of the MMA the photodetector signal was *V*_*o*_ = *R*_*s*_* G Ω P*_*o*_, where *R*_*s*_ is the responsively of photodetector, *G* is the transimpedance gain, *Ω* is a scale factor and *P*_*o*_ is the input light power to photodetector. The photodetector power was proportional to the reflected light intensity, *P*_*o*_ = *I*_*o*_*R*_*o*_, where *I*_*o*_ is the laser intensity at the surface of the MMA and *R*_*o*_ is the effective reflectance of the MMA. When SPM beads are on MMA their strong absorption and scatter behavior must be considered. If the reflectance of the MMA were uniform the opti-MMA signal without bead motion would be

2 $$V\left( t \right) = V_{o} - R_{s} G\Omega I_{o} (R_{o} - R_{p} )NA_{p}$$

where *R*_*p*_ is the *effective* reflectance of the *N* beads of area *A*_*p*_. The optical adsorption of the SPM beads will be determined by their physical properties, and we anticipate it will be proportional to their loading with iron oxide nanoparticles and follow a second order scale law with radius. The optical scattering of the SPM beads lies in the Mie scattering regime and thus also follow a second order scale law with radius,

In the phase-locked transport mode, the motion of the beads was described by a frequency dependent overlap parameter, *ζ(t)*, which was defined as the overlap of the beads with the micro-mirrors at different time points. In the case in which all beads moved in a synchronized phase-locked manner *ζ(t)* = *cos(ωt/2).* Thus, the value of *ζ(t)* is equal to 1 and 0 at *P1* (*θ*_*xz*_ = 0˚) and *P2* (*θ*_*xz*_ = 180˚), respectively, as shown in Fig. 1b. The output voltage of the photodetector for *N* beads on the MMA may expressed as

3 $$V\left(t\right)={V}_{o}-{R}_{s}G\varOmega N{A}_{p}{I}_{o}\left[\zeta \left(t\right){R}_{m}^{^{\prime}}-(1-\zeta \left(t\right)){R}_{Si}^{^{\prime}}\right]$$

where ${R}_{m}^{^{\prime}}$ and ${R}_{Si}^{^{\prime}}$ are the effective reflectivity of the micro-mirrors and silicon substrate, respectively, in the presence of the beads, i.e., ${R}_{Si}^{^{\prime}}$= *R*_Si_-*R*_*p*_. The minimum and maximum output voltages can be expressed in their simplest forms to be

4a $${V}_{min}={V}_{o}-{R}_{s}G\Omega N{A}_{p}{R}_{m}^{{\prime}}{I}_{o}, \;\text{at} \;\;\theta_{xz}=180^{\circ}, \;\text{and}$$

4b $${V}_{max}={V}_{o}-{R}_{s}G\Omega N{A}_{p}{R}_{Si}^{{\prime}}{I}_{o}, \; \text{at} \;\;\theta_{xz}=0^{\circ}.$$

Figure 3 present the result of the calculations of *V*_*min*_*/V*_*max*_ that were carried-out based Eq. (4). The empirical and materials parameters used for these calculations are listed in Table S1 (Supporting information). There is reasonably good agreement between the theoretical sensitivity and experimental observations for all four FOVs, i.e., *R*^2^ values are presented in the Fig. 3 caption. However, the model produced *V*_*min*_*/V*_*max*_ values that were consistently larger than the experimental results for FOVs 2–4. Figure 1d indicates that the light reflected from the MMA is not uniform. This suggests the position of the beads must be considered and would produce higher theoretical *V*_*min*_*/V*_*max*_ values.

**Figure 5:**
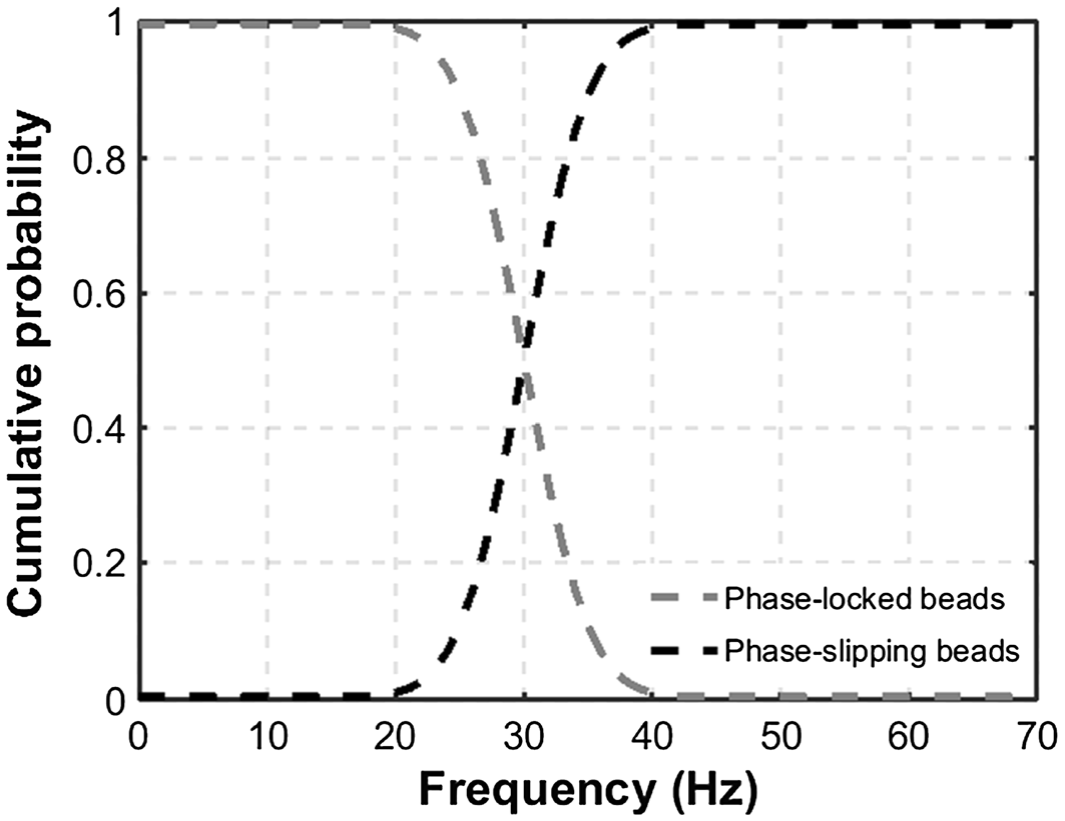
The theoretical population of phase-locked and phase-slipping beads as a function of the frequency of rotation of the external magnetic field. The probability of the SPM beads to enter the phase-slipping regime has previously been reported by this group and others to have a form consistent with the error function, e.g., Fig. 3a of Yellen et al.^21^.

At higher external field frequencies, *ω* > *ω*_*c*_, the motion of beads was not fully synchronized with external field. In this regime the frequency, the photodetector signal was determined by *ΔA* and the population of beads in the phase-slipping regime. Based on the analysis of the speed of a SPM bead in the NLM system we defined.

$$A=C((\omega -\sqrt{{\omega }^{2}-{\omega }_{c}^{2}})/{\omega }_{c}),$$

where *C* was a constant obtained empirically^21^. This behavior can be defined using a second overlap parameter

$$\psi (t)=((\omega -\sqrt{{\omega }^{2}-{\omega }_{c}^{2}})/{\omega }_{c})cos(\omega t/2)$$

for the fraction of beads in phase-slipping mode. The population of phase-locked and phase-slipping beads at certain oscillation frequencies could be determined using a cumulative distribution function which had been determined to have the form

5 $$N\left(\omega \right)=\left\{\begin{array}{ll}N; & \quad \omega \le {\omega }_{c}\\ \frac{N}{2}erfc \left(\frac{\left(\frac{\omega }{2\pi }\right)-\mu }{2\sqrt{\sigma }}\right); & \quad { \omega }_{c} < \omega \le {\omega }_{i}\\ 0; & \quad \omega > {\omega }_{i}\end{array}\right.$$

where *µ* is the mean and *σ* is the standard deviation obtained experimentally. Figure 5 presents the population of beads moving in a phase-locked and phase-slipping regime as a function of frequency.

Thus, the photodetector signal for *ω* > *ω*_*c*_ can be expressed in its simplest form in terms of *N(ω), ζ(t)* and $\psi \left(t\right)$

6 $$\begin{aligned} V\left( t \right) & = {V_o} - {R_s}G\varOmega N\left( \omega \right)\left( {{A_p} - \zeta \left( t \right)R_m^\prime {I_o} - {A_p}\left( {1 - \zeta \left( t \right)} \right)R_{Si}^\prime {I_o}} \right)\\ & \quad - {R_s}G\varOmega \left( {N - N\left( \omega \right)} \right)\left( {{A_p}\psi \left( t \right)R_m^\prime {I_o} - {A_p}\left( {1 - \psi \left( t \right)} \right)R_{Si}^\prime {I_o}} \right) \end{aligned}$$

Calculations of *V*_*min*_*/V*_*max*_ were carried-out as a function of *ω* based Eq. (6) and the results are presented as lines in Fig. 4. The materials parameters and constants used for calculation were listed in Table S1. The theoretical sensitivity of the opti-MMA system in the transport regime where the beads no longer are phase-locked and was in reasonable agreement with the experimental observations for all four FOVs.

Although this theoretical model produced a reasonable description of the response of the opti-MMA system, several assumptions have been made in its derivation. First, the incident laser light intensity, *I*_*o*_, in FOV was constant and homogenous. As we have already seen, variation in the laser intensity as a function of time and position will lead to systematic overestimation of the signal and increased noise. This can be corrected for a specific optical system. Second, we have assumed that the SPM beads adsorb and scatter light uniformly from the areas that they occupy on the MMA. This assumption had limited impact on the results presented in this study due to the strong optical activity of the beads, but obviously will need to be modified for smaller beads that may be bound to large analytes, such as, mammalian or bacterial cells. Third, we have assumed that we can accurately describe the SPM bead motion using Eq. (4), which is a function of the external magnet field. The primary sources of error in this assumption are the interactions of the beads with the MAA surface and each other that lead to transport behavior that is not consistent with Eq. (5). Nonspecific adhesion of the beads with the surface was observed for 1–5% of beads and these beads did not move under the external field and could not be detected in the *V*_*min*_*/V*_*max*_ values. The aggregation of beads also led to a significant change in transport behavior, i.e., the critical immobilization frequencies decreased significantly for aggregates^23^. The change in transport behavior resulting from bead aggregation can be observed in the optical signal at higher densities of beads in Fig. 4c for 90 beads on the MMA.

## Conclusion

This report analyzes the performance of an opti-MMA detection system that made it possible to simultaneously manipulate and detect individual SPM beads using a simple laser and photodetector detection system. The motion of 2.8 µm diameter SPM beads was found to strongly modulate the reflected light from the periodic micromagnets due to their optical properties. The difference in the effective reflectivity of the beads on the mirror like micromagnets and less reflective silicon surface produced a change in the total reflected light intensity that was synchronized with the motion of the beads at low frequencies. Above a critical frequency the beads entered a phase-slipping mode where the signal became decoupled from the rotation of the external magnetic field. The signal produced by the opti-MMA system has been defined in terms three classes of variables, i.e., optical properties of the detection system (e.g., FOV and detector and laser performance), materials properties (e.g., size and effective reflectivity of the beads), and transport of the SPM beads in the rotating external magnetic field.

The optical detection system signals, i.e., *V*_*o*_*, V*_*min*_ and *V*_*max*_, were proportional to the response of photodetector, the transimpedance gain, and the input light power to photodetector, which is dependent on both *P*_*o*_ and FOV. A small FOV produced higher bead sensitivity, *S*, while a larger FOV had a lower sensitivity due to the larger reflective area of the MMA relative to the size of a SPM bead. This meant that the signal-to-noise ratio (Δ*V*/Δ*V*_*noise*_) for the detector increased from 37, 10, 0.57 and 0 dB for FOV 1, 2, 3 and 4, respectively, where the noise amplitude was determined experimentally to be 1.5 mV. The sensitivity of the optical detection system made it possible to detect a single bead for FOV1/2, two beads for FOV3, and twenty-eight beads for FOV4.

The sensitivity of the optical detector was also closely related to physical properties of the MMA, SPM beads and the sample cell. The reflectance of the MMA (*R*_*o*_) is the sum of the relative reflectance of the micromagnets (${R}_{m}^{^{\prime}}$) and non-magnetic silicon substrate (${R}_{Si}^{^{\prime}}$), i.e., the relative intensity of the reflected light (Δ*V*/*V*_*max*_) should increase as ${R}_{Si}^{^{\prime}}$ decreases and ${R}_{m}^{^{\prime}}$ increases. This was confirmed for the substrate reflectance by producing an MMA on a transparent glass substrate. The sensitivity of the glass MMA device increased by a factor of 3.9 for the FOV3 optical configuration, i.e., *S* increased from 0.0007 to 0.0027 per bead (data not presented). This leads us to conclude that the sensitivity of the optical detection system can be increased sufficiently to detect individual 2.8 µm SPM beads in a field of view of > 40,000 µm^2^ by modification of the MMA optical properties. It is also theoretically possible to increase the sensitivity of the optical detection system by using smaller micromagnets.

The sensitivity of the detection scheme was also closely related to the size and effective reflectance of SPM bead used in the detection scheme. The optical signal should be proportional to the area of the bead, as shown in Eq. (3). The effective reflectance of the bead, however, will also be determined by their adsorption and scattering cross-sections. Both the optical absorption and Mie scattering cross-sections should be proportional to the cross-sectional area of the bead. Thus, the signal from the effective reflectance of smaller SPM beads is predicted to scale with the fourth order of the radius. However, measurements of the *V*_*min*_*/V*_*max*_ signal from 0.85 ± 0.2 µm diameter SPM beads that are composed of 70% by volume Fe_3_O_4_ nanoparticles^42^ were found to be only ~ 10 times smaller than the signal of the 2.8 µm SPM beads used in this study (data not presented). These results confirm that the optical signal decreases for smaller beads but suggest it scales like the second power of radius, although the optical adsorption of the smaller beads is quite a bit higher due it the higher loading of SPM nanoparticles. Obviously, there are other optical effects of relevance to the effective reflectance of SPM beads on the micromagnetic mirrors that are not accounted for in this simple model.

The highest sensitivity of the opti-MMA detection system was achieved when the SPM beads were transported in the phase-locked regime. A decrease in the sensitivity of the detection system will result from variations in the size, transport behavior, or aggregation state of the SPM beads. The physical properties of SPM beads can, however, be determined if the opti-MMA signal can be measured across a range of frequencies. For example, the optical signal could be measured at low frequencies to determine the number of beads in a FOV and then the frequency could then be increased, i.e., *ω*_*c*_ < *ω* < *ω*_*i*_*,* to determine the physical properties of the SPM bead-analyte complex, including size and magnetization. Such a measurement could be completed in a matter of seconds, as shown in Fig. 2.

In summary, the magnitude of an opti-MMA photodetector signal has been used to identify a single 2.8 µm diameter SPM bead in a field of view of 2,000 µm^2^ and in principle can be used to determine its size, magnetic properties and interaction with an analyte. We have also demonstrated that an optimized MMA design can be implemented to increase sensitivity to a single 2.8 µm bead over a 40,000 µm^2^ area or an 850 nm bead in a 49 µm^2^ area. In principle, such a system will allow as many as 10,0000 2.8 µm bead to be monitored per second using a FOV4 detection area and there is also the possibility of use multiple colors for multiplexing. These performance characteristics are similar to flow cytometry with the noted difference that the SPM bead transport is precisely controlled by magnetophoresis. These results and a recent bioanalytical demonstration^40^ lead us to conclude that optical-magnetophoresis provides a promising approach to rapid, cost-effectively, and sensitive detection of biological analytes with minimum consumption of reagents.

## Supplementary information

Supplementary Information.
