# Supplementary material for: Optical detection of the magnetophoretic transport of superparamagnetic beads on a micromagnetic array
Source: Sci Rep. 2020 Jul 30;10:12876. doi: 10.1038/s41598-020-69757-7 (PMC7392889; doi:10.1038/s41598-020-69757-7)
Supplement: Supplementary file 1 — Supplementary Information. [file 41598_2020_69757_MOESM1_ESM.docx]

**Optical detection of the magnetophoretic transport of superparamagnetic beads on micro-magnetic arrays**

Dhruv Gandhi, Peng Li*, Stefano Rampini, Charlotte Parent, and Gil Lee*

**Table S1**: Parameters and experimentally determined physical properties (italics) determined from linear regression of the least squared fit of the data presented in Figure 3.

| *Optical detection simulation parameters* | | | | |
| --- | --- | --- | --- | --- |
|  | DPSS 532-40 | | HL6322-G | |
| Input power (P) | 3 mW | | 3 mW | |
| Noise equivalent power (NEP) | 15X10^-13^ W/$\surd Hz$ | | 15X10^-13^ W/$\surd Hz$ | |
| Responsivity () | 0.32 A/W | | 0.4 A/W | |
| Bandwidth (*B*) | 5.6 kHz | | 5.6 kHz | |
| Transimpedence gain (*G*) | 4.75X10^6^ V/A | | 4.75X10^6^ V/A | |
| Input impedance | 10 G𝛺 in parallel with 100 pF | | 10 G𝛺 in parallel with 100 pF | |
| Scale factor | 1 | | 1 | |
| Temperature (*T*) | 298 K | | 298 K | |
| Noise fraction ($f_{I}$) | 0.0001 | | 0.0001 | |
| N.A. | 0.75 | 0.4 | 0.75 | 0.4 |
| Laser spot diameter ($1/e^{2}$) | *7 µm* | *25 µm* | *256 µm* | *726 µm* |
| Cobalt reflectance ($R_{m}$)* | *0.53±0.04* | *0.63±0.04* | *0.57±0.01* | *0.66±0.01* |
| Silicon reflectance ($R_{si}$)* | *0.22±0.02* | *0.34±0.02* | *0.24±0.01* | *0.33±0.01* |
| Bead reflectance ($R_{P}$)* | *0.018±0.001* | *0.030±0.002* | *0.018±0.0005* | *0.030±0.001* |

**Table S2**: Typical reflectance values in literature for comparison.

|  | | DPSS 532-40 | | HL6322-G | |
| --- | --- | --- | --- | --- | --- |
| Wavelength | | 532 nm | | 635 nm | |
| Numerical Aperture | | 0.75 | 0.4 | 0.75 | 0.4 |
| Reflectance^1^ | Cobalt | 0.5949 | 0.6374 | 0.6281 | 0.6683 |
|  | Silicon | 0.2969 | 0.3556 | 0.2783 | 0.3306 |
|  | Polystyrene | 0.028 | 0.046 | 0.028 | 0.046 |

1. M. N. Polyanskiy. "Refractive index database," http://refractiveindex.info (accessed June. 16 2016).
